# Generative AI, Cognitive Offloading, and Learner Agency in Higher Education: A Scoping Review

**DOI:** 10.3390/bs16071150

**Published:** 2026-07-08

**Authors:** Guanhua Wang, Wenna Wang, Daozhou Yang, Jifan Ren

**Affiliations:** 1 School of Economics and Management, Tsinghua University, Beijing 100084, China; wanggh@sem.tsinghua.edu.cn; 2 School of Economics and Management, Harbin Institute of Technology, Shenzhen 518055, China; wangwenna@hit.edu.cn; 3 Hubei Science and Technology Industry Information Research Center, Hubei University of Economics, Wuhan 430205, China

**Keywords:** generative artificial intelligence, learner agency, cognitive offloading, overreliance, self-regulated learning, higher education, scoping review

## Abstract

Generative artificial intelligence (GenAI) is increasingly integrated into higher education, where it supports writing, feedback, problem solving, and research-related tasks while also raising concerns about cognitive offloading and learner dependence. This scoping review maps the literature on the relationships among GenAI, cognitive offloading, and learner agency in higher education. Following PRISMA-ScR guidance, searches were conducted on 24 June 2026 across six database sources: Web of Science Core Collection, Scopus, EBSCOhost ERIC, EBSCOhost Education Source, APA PsycInfo via ProQuest, and ProQuest Education Collection via ProQuest Social Science Premium Collection. English-language peer-reviewed or scholarly journal literature published from 2022 to 24 June 2026 was eligible for inclusion. Data were charted using a structured extraction matrix and synthesized through descriptive mapping and theory-informed configurative synthesis. After duplicate removal, 5327 records were screened by title and abstract, 691 full-text reports were assessed, and 123 studies were included in the final charting matrix and synthesis. The review shows that learner agency is conceptualized as a multidimensional construct involving self-regulation, reflective judgement, intentionality, and responsible action. Across the literature, GenAI is best interpreted through an interpretive dual-pattern account: agency-supportive patterns are associated with self-regulated learning, self-efficacy, feedback literacy, and reflective engagement, whereas agency-eroding patterns are associated with cognitive offloading, overreliance, dependence, uncritical uptake, and weakened judgement. These patterns should be interpreted as a configurative synthesis of heterogeneous evidence rather than as causal effect estimates. Overall, the findings suggest that the educational value of GenAI depends less on the technology itself than on how it is pedagogically embedded, with augmentation-oriented and scaffolded use being more supportive of learner agency than replacement-oriented use.

## 1. Introduction

Generative artificial intelligence (GenAI) is rapidly reshaping learning practices in higher education. Across a wide range of academic tasks, including writing, reading, feedback uptake, problem solving, and research support, GenAI has increasingly become part of students’ everyday learning environments. Existing studies suggest that GenAI can provide immediate feedback, personalized assistance, adaptive dialogue, and content generation, thereby extending traditional forms of academic support and creating new possibilities for learner-centered education (Zhan & Yan, 2025; R. Zhou et al., 2025; Zhu & Yang, 2026).

At the same time, the educational implications of GenAI are increasingly described in contradictory terms. On the one hand, a growing body of research reports that GenAI can strengthen engagement, improve perceived competence, enhance self-efficacy, and support writing, feedback, and research performance (Q. Zhang et al., 2025; C. Zhou & Wang, 2026; Zhu & Yang, 2026). On the other hand, scholars have also raised serious concerns about technological dependence, uncritical acceptance of AI-generated output, diminished originality, and the erosion of independent judgement. Some studies now describe this pattern as paradoxical: GenAI may simultaneously increase learners’ confidence and efficiency while also intensifying their dependence on technology (L. Zhang & Xu, 2025; Zheng & Wang, 2026).

These tensions suggest that it is no longer sufficient to evaluate GenAI merely in terms of learning outcomes, performance gains, or technology acceptance. A more analytically useful lens is learner agency. Recent studies have begun to conceptualize learner agency in GenAI-supported contexts as a multidimensional construct rather than a vague ideal. For example, L. Xia et al. (2025) defined student learning agency in GenAI-supported contexts in terms of key abilities, active actions, and essential mental characteristics. Similarly, Yang et al. (2024) proposed that student agency in GenAI-mediated higher education can be understood through different modes of engagement, including receptive, resistive, resourceful, and reflective learning activities. Together, these studies suggest that the key issue is not whether students use GenAI but whether they remain able to direct, monitor, evaluate, and take responsibility for that use as active learners (L. Xia et al., 2025, 2026; Yang et al., 2024).

From this perspective, the relationship between GenAI use and learner agency appears to be associated with a recurring cluster of cognitive, motivational, and socio-technical mechanism-related constructs. Self-regulation and metacognition are especially prominent in the reviewed literature, but they are not treated here as a formally ranked causal hierarchy. Rather, studies repeatedly show that when learners engage in goal setting, monitoring, evaluation, verification, and strategic revision, GenAI is more likely to function as a scaffold for learning than as a substitute for thinking. By contrast, when such regulatory capacities are weak, learners are more likely to slide into superficial engagement, passive uptake, and cognitive offloading (Xu et al., 2025; Zheng & Wang, 2026; C. Zhou & Wang, 2026). Related studies also point to the important roles of trust, self-efficacy, anxiety, AI literacy, and feedback literacy in shaping how learners interpret and use GenAI support (Zhan & Yan, 2025; Q. Zhang et al., 2025; C. Zhou & Wang, 2026).

Despite this growing body of work, the field remains fragmented in several ways. First, many studies still focus on isolated variables such as intention to use, satisfaction, writing performance, or self-efficacy, without systematically examining learner agency as the broader organizing construct. Second, although overreliance, dependence, and cognitive offloading are repeatedly mentioned in the literature, they are rarely integrated into a unified account of how GenAI may undermine learner agency. Third, the literature is methodologically and contextually heterogeneous, spanning writing, reading, speaking, feedback, problem solving, and research tasks, and employing surveys, experiments, qualitative methods, mixed-methods designs, and scale development studies. This heterogeneity makes a scoping review especially appropriate for mapping the field and clarifying its conceptual and empirical structure (L. Xia et al., 2025, 2026; Zhu & Yang, 2026).

Accordingly, this review argues that the educational significance of GenAI in higher education should be understood through an interpretive dual-pattern perspective. One pattern points toward agency support through stronger self-regulation, greater confidence, more active feedback engagement, and deeper participation in learning. The other points toward agency erosion through cognitive offloading, overreliance, technological dependence, and weakened independent judgement. Crucially, these patterns do not arise automatically from the technology itself; rather, they depend on how GenAI is used, how learners regulate that use, and whether pedagogical designs preserve reflection, evaluation, and responsibility (Wang & Zhang, 2026; L. Zhang & Xu, 2025; Zheng & Wang, 2026).

Against this background, the present study conducts a scoping review of research on GenAI, cognitive offloading, and learner agency in higher education. Specifically, this review aims to examine how learner agency has been conceptualized in GenAI-supported learning, how GenAI may be associated with both agency-supportive and agency-eroding patterns, which recurring constructs and proposed mechanisms help explain how GenAI use is associated with agency-related outcomes, and which pedagogical conditions help ensure that GenAI functions as augmentation rather than replacement. By doing so, the review seeks to provide a theory-informed configurative framework for understanding more reflective, responsible, and agentic uses of GenAI in higher education (L. Xia et al., 2026; C. Zhou & Wang, 2026; Zhu & Yang, 2026).

## 2. Conceptual Background

### 2.1. Learner Agency in GenAI-Supported Higher Education

Learner agency has become an increasingly important concept for understanding how students act within GenAI-supported learning environments. In the present review, learner agency is not treated as a vague synonym for autonomy or participation. Rather, it is understood as learners’ capacity to purposefully direct, monitor, evaluate, and take responsibility for their own learning while interacting with GenAI tools. This means that agency involves more than simply using AI; it concerns whether learners remain the ones who set goals, make judgements, select strategies, and regulate the learning process. In this sense, learner agency provides a more useful analytical lens than outcome indicators alone because it captures how students position themselves in relation to AI-supported learning rather than only what they achieve after using it (L. Xia et al., 2025, 2026).

Recent studies have begun to operationalize learner agency in more explicit and measurable ways. L. Xia et al. (2025), for example, developed a student learning agency scale specifically for GenAI-supported contexts and proposed that learner agency consists of three interrelated dimensions: key abilities, active actions, and essential mental characteristics. Within this structure, self-regulation and self-reflection are treated as foundational abilities; motivation, self-efficacy, and volition are treated as central psychological characteristics; and participation, selectivity, and responsibility are treated as core expressions of agentic action. This multidimensional account is important because it shows that agency in AI-supported learning is simultaneously cognitive, motivational, and behavioral. It is therefore insufficient to infer agency from tool use alone or from positive attitudes toward AI. Instead, agency must be examined through the ways learners interpret, control, and act upon AI support in the course of learning (L. Xia et al., 2025).

A complementary perspective is offered by Yang et al. (2024), who framed student agency in GenAI-supported higher education as a movement from surface to deep learning. Their analytical framework distinguishes among receptive, resistive, resourceful, and reflective forms of student activity. This distinction is especially useful for the present review because it makes clear that students may engage with GenAI in qualitatively different ways. Some may simply receive or reproduce AI output, whereas others may challenge, adapt, extend, or reflect upon it. Thus, GenAI use does not automatically imply either passivity or empowerment; what matters is the form of engagement through which learners exercise judgement and control. In this respect, learner agency is best seen as a continuum of increasingly active and reflective participation rather than as a binary attribute that learners either possess or lack (Jiang et al., 2026; Lai et al., 2026; Yang et al., 2024).

Taken together, these studies suggest that learner agency should serve as the organizing construct for interpreting GenAI use in higher education. It allows the field to move beyond narrow questions of acceptance, frequency of use, or task completion and toward a more meaningful question: whether learners remain capable of directing their own learning in AI-mediated contexts. This is particularly important because the same technology may support agentic engagement in one context while fostering passive dependence in another. A conceptually robust account of learner agency is therefore necessary for distinguishing between these divergent possibilities (L. Xia et al., 2025; Yang et al., 2024).

### 2.2. Cognitive Offloading, Overreliance, and Dependence

If learner agency captures the possibility of purposeful and reflective AI-supported learning, then cognitive offloading represents its critical counterpoint. Broadly speaking, cognitive offloading refers to the delegation of cognitive work to external tools or systems. In the context of GenAI, this may include outsourcing idea generation, drafting, summarizing, revising, decision-making, or evaluative judgement to the AI system. Importantly, the literature suggests that offloading is not inherently maladaptive. Some forms of strategic offloading may reduce unnecessary load, free up cognitive resources, and support higher-order thinking. However, the same process may also become problematic when learners rely on GenAI without verification, without reflection, or without maintaining ownership of the task (Chase & Galvin, 2026; Wang & Zhang, 2026).

This distinction between adaptive and maladaptive offloading is increasingly explicit in recent research. Wang and Zhang (2026) proposed that pedagogical partnerships with GenAI may activate two cognitive pathways simultaneously: vigilance and offloading. Their account is especially valuable because it avoids assuming that all offloading is negative. Instead, they argue that strategic offloading may, under appropriate conditions, support transformative learning by reallocating effort toward more advanced reflection. Yet they also show that such benefits depend on whether learners preserve critical oversight. In contrast, Chase and Galvin (2026) emphasized the risk of “outsourcing to GenAI” in ways that undermine learning validity and weaken cognitive ownership. Together, these studies indicate that the central issue is not whether learners offload at all but whether they offload in ways that remain subordinate to reflection, judgement, and educational purpose.

Empirical studies further show that overreliance is not a single undifferentiated phenomenon. Hou et al. (2025), for instance, distinguished among reflective, cautious, thoughtless, and collaborative reliance behaviors when undergraduates used GenAI for problem solving. Their findings suggest that reliance becomes problematic when trust is not balanced by critical thinking and when learners shift from reflective or cautious use toward thoughtless use. Similarly, Zheng and Wang (2026) identified different patterns of cognitive dissonance in GenAI-supported learning, including efficiency–capacity dissonance and trust–reliance dissonance, showing that learners often experience productivity gains and dependency concerns at the same time. These studies reinforce the idea that overreliance is better understood as a patterned behavioral and psychological response than as mere frequency of use (Hou et al., 2025; Zheng & Wang, 2026).

The paradox becomes even clearer in studies that examine dependence and self-belief together. L. Zhang and Xu (2025) found that more frequent GenAI use could simultaneously enhance students’ confidence and efficiency while intensifying technological dependence. Likewise, Jia et al. (2025) showed that GenAI dependency may distort academic self-evaluation, creating a form of false self-efficacy associated with cognitive outsourcing. These findings are particularly important for the present review because they show why agency cannot be inferred from confidence alone. Higher self-efficacy in AI-mediated learning may sometimes reflect genuine competence, but it may also coexist with reduced independent capability when learners come to rely on AI as an indispensable substitute rather than a supportive tool (L. Zhang & Xu, 2025; Jia et al., 2025).

For this reason, cognitive offloading, overreliance, and dependence are treated in this review as important agency-eroding/offloading patterns through which learner agency may be weakened. They represent the possibility that GenAI use, rather than enhancing learners’ capacity to direct their own thinking, may gradually transfer control over judgement, strategy, and evaluation from the learner to the system. Understanding this agency-eroding/offloading pattern is essential if GenAI is to be conceptualized not simply as a helpful educational tool but as a technology whose educational implications depend on how responsibility for thinking is distributed between human learners and AI systems (Wang & Zhang, 2026; L. Zhang & Xu, 2025; Zheng & Wang, 2026).

To further ground this distinction, cognitive offloading can also be viewed through the lens of technological cognition. From this perspective, technology use does not merely reduce workload; it redistributes cognitive operations between learner and tool. In GenAI-supported learning, the key question is therefore which operations remain actively performed by the learner, such as goal setting, verification, causal or epistemic reasoning, and evaluative judgement, and which are displaced to the system. This perspective helps distinguish strategic offloading, in which external support remains subordinate to learner oversight, from maladaptive offloading-as-displacement, in which the system substitutes for learner reasoning (Federico et al., 2026; Bluet et al., 2025).

### 2.3. Self-Regulation as a Recurring Linking Construct

Self-regulation recurs across the reviewed literature as a key construct linking GenAI use with agency-related outcomes. Its importance lies in the fact that GenAI does not act on learners in a direct, deterministic way. Instead, reported learning patterns are filtered through the extent to which learners can set goals, monitor progress, evaluate outputs, revise strategies, and regulate their own motivation and attention while using the technology. In other words, self-regulation helps explain why the same GenAI affordances may be associated with deeper learning for some students but superficial completion or dependence for others (L. Xia et al., 2025; Xu et al., 2025).

This recurring role of self-regulation is supported by recent studies on metacognitive support. Xu et al. (2025) found that when learners worked in GenAI environments without explicit metacognitive support, their self-regulated learning could decline; by contrast, when such support was introduced, learners showed stronger task strategy use, self-evaluation, and overall learning experience. Related studies similarly indicate that effective GenAI use depends heavily on metacognitive awareness. Teng (2025) showed that students with stronger metacognitive awareness were more likely to use ChatGPT as a feedback and revision resource, whereas weaker metacognitive awareness was associated with direct copying or shallow uptake. These findings indicate that self-regulation is not merely an additional benefit of AI use; it is one of the conditions under which GenAI becomes educationally valuable in the first place (Xu et al., 2025; Teng, 2025).

At the same time, self-regulation rarely operates alone. It is closely intertwined with trust, self-efficacy, anxiety, and feedback literacy. For example, C. Zhou and Wang (2026) found that generative AI acceptance positively predicted writing feedback literacy, both directly and indirectly through higher writing self-efficacy and lower writing anxiety. Q. Zhang et al. (2025) similarly showed that technological features such as responsiveness and personalization enhanced learning engagement through the mediating roles of AI trust and AI learning self-efficacy. These studies suggest that self-regulation in GenAI-supported learning is embedded in a broader psychological system: learners regulate more effectively when they trust the tool appropriately, feel capable of using it, and are less emotionally overwhelmed by the task.

The literature also indicates that self-regulation is deeply tied to evaluative judgement and critical thinking. Urban et al. (2025) demonstrated that epistemic beliefs and metacognitive accuracy influenced whether students integrated correct or incorrect ChatGPT content into their writing. Zhu and Yang (2026), in turn, showed that the re-lationship between GenAI use and graduate students’ research competence was mediated by critical thinking and moderated by research self-efficacy. These findings are especially important because they show that self-regulation in GenAI environments is not limited to effort management or planning; it also includes epistemic monitoring, verification, and the ability to resist the automatic acceptance of plausible AI output.

For the purposes of this review, self-regulation is therefore treated as a recurring and integrative linking construct between GenAI use and learner agency rather than as a single causal mechanism established by one uniform body of evidence. When supported by metacognitive awareness, critical thinking, calibrated trust, AI literacy, and self-efficacy, self-regulation enables learners to use GenAI as a scaffold for agency. When these conditions are absent, GenAI use may shift toward cognitive offloading, uncritical uptake, and dependence. This interpretation provides the basis for the dual-pathway framework developed in the review, while remaining cautious about the heterogeneous and mostly non-causal evidence base.

## 3. Methods

### 3.1. Review Design

This study adopted a scoping review design to map the emerging literature on generative artificial intelligence (GenAI), cognitive offloading, and learner agency in higher education. A scoping review was considered appropriate because the field is conceptually diverse and methodologically heterogeneous, spanning qualitative, quantitative, mixed-methods, experimental, conceptual, review, and scale-development studies across multiple educational contexts. Rather than estimating a single pooled effect, the purpose of the review was to identify how learner agency has been conceptualized, how GenAI has been linked to agency-supportive and agency-eroding patterns of use, and which mechanism-related constructs and pedagogical conditions have been proposed in the literature. The review is therefore best understood as a PRISMA-ScR-informed scoping review with descriptive mapping and theory-informed configurative synthesis, not as a meta-analysis or a fully inductive thematic analysis. This approach is consistent with the foundational rationale of scoping reviews as a method for mapping key concepts, research trends, and knowledge gaps in developing fields (Arksey & O’Malley, 2005; Levac et al., 2010; Munn et al., 2018).

The review process was further informed by the PRISMA-ScR reporting framework to ensure transparency in searching, screening, data charting, and synthesis (Tricco et al., 2018); the completed PRISMA-ScR checklist is provided in Appendix A.

The protocol for this scoping review was registered in INPLASY. The registration number is INPLASY202650152, and the protocol is available at 10.37766/inplasy2026.5.0152.

Bibliographic records, screening decisions, data-charting matrices, and Supplementary Tables S1 and S2 were managed using Microsoft Excel (Version 16.110.2). Inter-rater agreement statistics, including Fleiss’ kappa and pairwise Cohen’s kappa, were calculated using Python (Version 3.12.13).

### 3.2. Search Strategy

The updated literature search was conducted on 24 June 2026 across six database sources available through the university library: Web of Science Core Collection, Scopus, EBSCOhost ERIC, EBSCOhost Education Source, APA PsycInfo via ProQuest, and ProQuest Education Collection via ProQuest Social Science Premium Collection. These databases were selected to improve coverage across education, psychology, interdisciplinary social science, applied linguistics, and technology-related higher education research.

The search strategy was organized around three conceptual blocks: (1) GenAI technologies, including ChatGPT, generative AI, generative artificial intelligence, GenAI, large language models, LLMs, GPT, GPT-3, and GPT-4; (2) higher education contexts, including higher education, university, college, tertiary education, undergraduate, postgraduate, graduate student, university student, and college student; and (3) agency-, offloading-, and mechanism-related constructs. The third block included learner agency, student agency, learning agency, autonomy, self-directed learning, cognitive offloading, overreliance, AI reliance, dependence, technological dependence, cognitive outsourcing, automation bias, self-efficacy, self-regulation, metacognition, critical thinking, feedback literacy, trust, evaluative judgement/judgment, and AI literacy.

To address the recall limitations of the earlier single-database, title-focused strategy, GenAI-related terms were searched in title, abstract, keyword, topic, subject, NOFT, or database-equivalent indexed fields according to each platform. The search was limited to English-language peer-reviewed or scholarly journal literature published from 2022 to 24 June 2026. No exclusion terms for primary education, secondary education, clinical populations, or non-student contexts were built into the database search strings; these exclusions were instead applied during screening to avoid accidentally removing relevant higher education studies. Full database-specific search strings and filters are provided in Appendix B.

### 3.3. Eligibility Criteria

Studies were included if they met all of the following criteria:

1. They were peer-reviewed or scholarly journal articles, including articles, review articles, and early-access articles, where allowed by the database filter.
2. They focused on GenAI tools, such as ChatGPT or other large language model-based systems.
3. They were situated in higher education contexts, including undergraduate, postgraduate, or other tertiary-level learning settings.
4. They provided substantive evidence, conceptual analysis, or operationalization of at least one construct directly related to learner agency, autonomy, self-directed learning, cognitive offloading, overreliance, dependence, AI reliance, self-regulation, metacognition, critical thinking, feedback literacy, self-efficacy, trust, AI literacy, evaluative judgement, or related mechanisms in higher education students’ learning processes. Keyword presence alone was not sufficient for inclusion.
5. They were published in English.

Studies were excluded if they were editorials, commentaries, opinion essays, conference abstracts, theses, book chapters, non-peer-reviewed reports, or records without sufficient retrievable bibliographic information. Studies were also excluded when they focused primarily on K-12 education, clinical or patient populations, teacher/faculty adoption, institutional governance, AI detection, technical system performance, performance/satisfaction/attitudes only without an analyzable agency/offloading mechanism, or general technology acceptance without direct relevance to higher education students’ learning processes or agency/offloading-related mechanisms.

Because widespread educational use of GenAI emerged after the public release and rapid uptake of ChatGPT and related tools, the review focused on literature published from 2022 onward. The search endpoint was 24 June 2026.

### 3.4. Study Selection

All retrieved records were exported from the six database sources and screened in a staged process. The searches identified 8020 records. After 2693 duplicate records were removed, 5327 records remained for title and abstract screening. Three authors independently screened the title and abstract records against the eligibility criteria after calibration on a pilot subset. Disagreements and uncertain cases were discussed, and unresolved records were adjudicated by a fourth author. For the title/abstract stage, three-category include/unsure/exclude coding showed substantial overall agreement (Fleiss’ kappa = 0.7103; pairwise Cohen’s kappa = 0.7147, 0.5799, and 0.8309). This process led to 4610 title/abstract exclusions and 717 reports sought for full-text retrieval.

**Figure 1:**
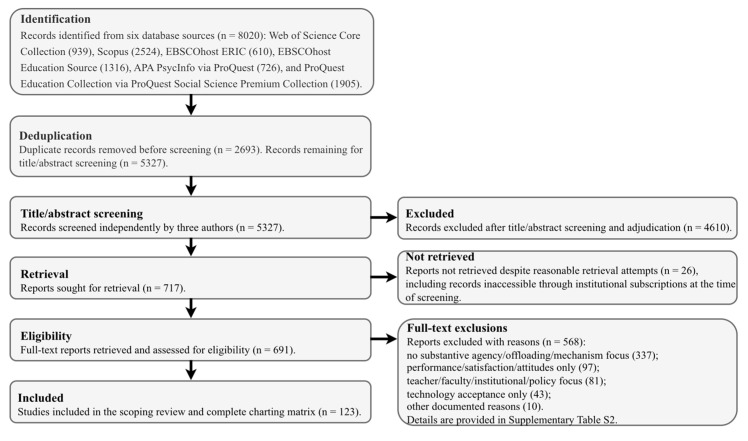
PRISMA-ScR flow diagram of study selection, showing database retrieval, duplicate removal, independent title/abstract screening, fourth-author adjudication, full-text retrieval status, full-text exclusions with reasons, and final inclusion.

Of the 717 reports sought for retrieval, 26 could not be retrieved despite reasonable retrieval attempts through institutional database links, publisher pages, DOI landing pages, interlibrary or library-accessible options, and other accessible full-text routes. These unretrieved reports included records for which full text was not accessible through the authors’ institutional subscriptions at the time of screening. Therefore, 691 full-text reports were retrieved and assessed for eligibility. Three authors then independently rescreened the 691 full texts using a binary include/exclude decision, followed by consensus discussion and fourth-author adjudication. Initial full-text agreement varied across author pairs, ranging from fair to substantial (Fleiss’ kappa = 0.4884; pairwise Cohen’s kappa = 0.3939, 0.3056, and 0.8475). This variation reflected the interpretive boundary between broad GenAI-in-education relevance and substantive relevance to learner agency, cognitive offloading, reliance/dependence, or mechanism-related learning processes. All disagreements were therefore resolved through consensus discussion, with unresolved cases adjudicated by the fourth author. After adjudication, 568 reports were excluded with reasons and 123 studies were included in the final charting matrix and synthesis. The main full-text exclusion reasons were: general GenAI in higher education but no substantive focus on learner agency/offloading mechanisms (n = 337); performance, satisfaction, or attitudes only without an analyzable agency/offloading mechanism (n = 97); teacher, faculty, institutional, policy, or administrative focus without student learning process evidence (n = 81); technology acceptance or intention-to-use without direct student learning process or agency-related evidence (n = 43); insufficient analyzable evidence or conceptual content (n = 7); not GenAI/LLM-based after full-text review (n = 2); and wrong source type or document type after full-text verification (n = 1). Detailed full-text exclusion reasons are reported in Supplementary Table S2. The complete study selection process is summarized in Figure 1.

### 3.5. Data Charting

A structured data-charting form was developed to extract both bibliographic information and substantive review data from the included studies. The substantive charting fields were prioritized because the purpose of the review was to map how GenAI, learner agency, cognitive offloading, reliance/dependence, and related mechanisms were conceptualized and studied.

- The charting form included the following fields: study ID; author/year; title; journal/source; DOI; database source; country/region where identifiable; discipline or educational context; participant group; GenAI tool/platform; learning task or activity; study design/method; evidence category; learner agency definition or proxy construct; cognitive offloading, reliance, overreliance, or dependence construct; agency-supportive evidence; agency-eroding/offloading evidence; mechanism-related constructs, mediators, or moderators; pedagogical boundary conditions; theme assignment; inclusion rationale; and charting notes.
- Additional audit fields were retained to support transparency and reproducibility, including database export file, document type, metadata-verification status, full-text relevance score, synthesis role, file-manifest links, and title/author/year/DOI/source verification notes.

This charting strategy was designed to support both descriptive mapping and conceptual synthesis. The complete charting matrix for the 123 included studies is provided as Supplementary Table S1. The matrix records bibliographic details, database source, document type, evidence category, study design/method, country/region where identifiable, educational context, participant group, GenAI tool/platform, focal constructs, learner-agency and offloading/reliance/dependence constructs, mechanism codes, agency-supportive and agency-eroding/offloading evidence, boundary conditions, theme assignment, inclusion rationale, synthesis role, verification status, and file-manifest fields. Bibliographic metadata in Supplementary Table S1 were cross-checked against the original six database export files; all 123 included-study records had complete title, author, year, DOI, and journal/source fields after verification.

### 3.6. Data Synthesis

The 123 included studies were synthesized through descriptive mapping and theory-informed configurative synthesis. First, the literature was mapped in terms of publication year, database source, document type, evidence type, and charted construct signals. Second, the charted findings were organized through a framework synthesis logic: the initial sensitizing framework distinguished agency-supportive and agency-eroding/offloading patterns, while repeated reading of the included studies was used to refine the mechanism-related construct categories and boundary conditions.

Coding proceeded in four steps. First, each included study was coded for evidence type and document/source characteristics. Second, each study was coded for whether it contributed charted evidence to agency/autonomy, offloading/reliance/dependence, and mechanism-related constructs. Third, mechanism codes were reviewed across studies and consolidated into recurrent clusters, including self-regulation, metacognition, critical thinking, self-efficacy, trust, feedback literacy, AI literacy, and cognitive offloading/reliance/dependence. Fourth, the thematic structure was checked against the full charting matrix to ensure that conceptual claims were not treated as equivalent to empirical causal evidence.

For coding purposes, a charted construct signal was recorded only when a construct was substantively discussed, measured, operationalized, or used to interpret findings; keyword occurrence alone did not count. Construct signals indicate relevance to a conceptual domain, whereas theme-family counts indicate that the study contributed evidence or conceptual material to a synthesized result theme. Agency-supportive and agency-eroding/offloading contributions were coded when the charted evidence addressed reflective, self-regulated, evaluative, or scaffolded uses of GenAI, or, conversely, cognitive offloading, overreliance, dependence, uncritical uptake, weakened judgement, or displacement of learner responsibility. We calibrated the charting form on an initial subset, discussed code definitions, and resolved uncertain charting decisions by consensus; disputed cases were reviewed by the fourth author.

1. How learner agency is conceptualized in GenAI-supported higher education;
2. Positive pathways through which GenAI may enhance learner agency;
3. Negative pathways involving cognitive offloading, overreliance, and dependence;
4. Recurring mechanism clusters linking GenAI use to agency-related outcomes, especially self-regulation, metacognition, critical thinking, self-efficacy, trust, feedback literacy, AI literacy, and cognitive offloading/reliance/dependence;
5. Boundary conditions and pedagogical responses that shape whether GenAI functions as augmentation or replacement.

This synthesis strategy was chosen because the field contains substantial conceptual variation and does not yet support narrow effect-size aggregation. The resulting framework should therefore be read as an interpretive synthesis of heterogeneous evidence rather than as a causal model or a ranking of mechanisms by evidential strength. Where the manuscript refers to recurring mechanisms, the claim is that these constructs appeared repeatedly across the charted corpus, not that formal critical appraisal or meta-analysis established their comparative causal importance.

## 4. Results

The updated evidence base consisted of 123 included studies that met the full eligibility criteria and had a substantive connection to GenAI, higher education learners, and learner agency, cognitive offloading, reliance/dependence, or related mechanisms. Table 1 summarizes the characteristics of the included studies and Supplementary Table S1 provides the complete verified charting matrix.

As shown in Table 1 and Supplementary Table S1, descriptive mapping showed that the included literature was concentrated in recent higher education research on writing, feedback, problem solving, research training, language learning, and self-regulated learning contexts. Country/region, educational-context, participant-group, and task/activity fields were coded as non-mutually exclusive charting signals because a single study could involve multiple contexts, countries, participant groups, or learning activities. The most frequent identifiable country/region signals were China (n = 58) and the United States (n = 46), while 15 studies did not have a clearly identifiable country/region in the available charting fields. Context signals were especially common for research/postgraduate learning (n = 100), discipline-specific higher education (n = 77), language/EFL/ESL contexts (n = 71), writing/feedback (n = 70), and STEM/problem-solving contexts (n = 70). Participant-group signals most often involved undergraduate/university students (n = 91), EFL/ESL learners (n = 61), and postgraduate/graduate students (n = 40). The charted evidence base includes empirical studies, review/evidence-synthesis articles, and conceptual/framework sources. These sources were not treated as carrying identical evidential weight: empirical findings were used to map observed learning processes, while review and conceptual/framework sources were used primarily to clarify constructs, mechanism-related patterns, and boundary conditions. Theoretical sources cited in the Discussion for broader conceptual framing, such as work on technological cognition, are not counted as included studies unless they met the review eligibility criteria and appear in Supplementary Table S1.

### 4.1. Conceptualizations of Learner Agency in GenAI-Supported Higher Education

Of the 123 included studies, 86 contributed to the theme family on learner agency/autonomy conceptualization, while agency/autonomy construct signals were recorded in 121 studies. The literature reviewed in this study shows that learner agency in GenAI-supported higher education is conceptualized in multiple, partially overlapping ways rather than as a single, universally agreed construct. At the broadest level, learner agency is commonly understood as students’ capacity to act intentionally, regulate their goals and strategies, evaluate AI-supported outputs, and influence the direction of their own learning rather than merely responding passively to external support. In this sense, agency is closely related to self-regulation, reflective judgement, and the ability to remain the active organizer of learning in AI-mediated environments (L. Xia et al., 2025, 2026).

A particularly important conceptualization is offered by L. Xia et al. (2025), who developed a student learning agency scale for GenAI-supported contexts and proposed a three-dimensional framework consisting of key abilities, active actions, and essential mental characteristics. Within this structure, agency includes foundational abilities such as self-regulation and self-reflection, motivational and volitional characteristics such as self-efficacy and persistence, and action-oriented expressions such as participative, selective, and responsible engagement. This framework is significant because it moves the field beyond loosely defined notions of autonomy and provides a more systematic way to understand agency as simultaneously cognitive, motivational, and behavioral (L. Xia et al., 2025).

A complementary perspective is provided by Yang et al. (2024), who conceptualized learner agency through the distinction between receptive, resistive, resourceful, and reflective forms of engagement with GenAI. This framework is especially useful because it captures variation in how students interact with AI support. Some learners receive and reproduce GenAI outputs with limited reflection, whereas others question, adapt, extend, or critically evaluate them. In this sense, learner agency is not simply a trait that students possess but a mode of engagement that becomes visible through their interactional patterns with GenAI-supported tasks (Yang et al., 2024).

Other studies conceptualize learner agency more specifically through revision, self-directed learning, or co-writing control. For example, Jiang et al. (2026) framed agency in revising GenAI-generated statements of purpose as the learner’s ability to evaluate, reshape, and take ownership of AI-generated text, identifying patterns ranging from compliance-oriented acceptance to content-oriented innovation. Similarly, Kim et al. (2026) conceptualized agency in collaborative writing as intentionality and self-monitoring and demonstrated that interface features can be explicitly designed to help learners remain “in the loop” rather than deferring uncritically to AI suggestions. Lai et al. (2026), from a more relational perspective, extended the concept further by proposing dimensions such as proxy, collective, and shared agency, suggesting that agency may also emerge through how students coordinate human and technological resources across learning environments (Jiang et al., 2026; Kim et al., 2026; Lai et al., 2026).

Taken together, these studies indicate that learner agency in GenAI-supported higher education should be understood as a multidimensional and context-sensitive construct. It involves learners’ ability to maintain intentionality, evaluative judgement, and responsibility while interacting with GenAI. This conceptualization is central to the present review because it provides a framework for understanding why GenAI may sometimes enhance students’ learning capacity while, in other cases, contributing to dependency, passivity, or reduced ownership over learning.

### 4.2. Agency-Supportive Patterns: How GenAI May Support Learner Agency

Of the 123 included studies, 118 contributed to the agency-supportive theme family. A substantial portion of the literature portrays GenAI as a resource that can strengthen learner agency under appropriate conditions. Across writing, reading, problem-solving, research, and feedback contexts, studies repeatedly report that GenAI may support learners’ autonomy, self-direction, engagement, and confidence by making academic assistance more immediate, personalized, and interactive. In many studies, these agency-supportive patterns are not interpreted as simple technological gains but as evidence that students can become more active and capable learners when AI support is embedded in ways that preserve decision-making and reflection (L. Xia et al., 2026; Q. Zhang et al., 2025; R. Zhou et al., 2025).

One agency-supportive pattern concerns the development of self-regulated learning. Multiple studies suggest that GenAI can facilitate goal setting, planning, monitoring, and self-evaluation when learners use it as a learning scaffold rather than as a substitute for task completion. Yilmaz et al. (2026), for instance, found that GenAI-supported feedback helped improve online learners’ regulation skills, especially among students with initially lower levels of self-regulated learning. Similarly, studies on writing and feedback suggest that when learners interact actively with AI-generated prompts or dialogic feedback, they become more likely to engage in revision, reflection, and strategic uptake of feedback (Yilmaz et al., 2026; C. Zhou & Wang, 2026; Zhan & Yan, 2025).

A second agency-supportive pattern concerns self-efficacy. A wide range of studies report that GenAI use may increase learners’ confidence in their ability to complete academic tasks, especially when AI support reduces uncertainty, offers immediate responses, or helps learners structure complex tasks. For example, Q. Zhang et al. (2025) found that responsiveness and personalization enhanced engagement partly by increasing AI learning self-efficacy, while Zhu and Yang (2026) showed that GenAI use was positively associated with graduate students’ research competence, with research self-efficacy functioning as an important moderator. In these studies, self-efficacy operates as a psychological bridge through which students perceive themselves as more capable participants in AI-supported learning, rather than as passive users of automated tools (Q. Zhang et al., 2025; Zhu & Yang, 2026).

A third agency-supportive pattern involves feedback literacy and evaluative participation. In GenAI-supported writing environments, several studies show that students can use AI-generated feedback to become more active readers, interpreters, and users of feedback. C. Zhou and Wang (2026) found that generative AI acceptance positively predicted writing feedback literacy both directly and indirectly through higher writing self-efficacy and lower writing anxiety. Related work also shows that structured reflection, strategic prompting, and dialogic feedback can help students better understand feedback, judge its value, and apply it more selectively. From an agency perspective, these findings are important because they suggest that GenAI can support not only task performance but also students’ capacity to engage with feedback more deliberately and productively (C. Zhou & Wang, 2026; Zhan & Yan, 2025).

Finally, agency-supportive patterns are also visible in studies of research competence, reading support, and problem solving. Zhu and Yang (2026) reported that different levels of GenAI use were positively associated with graduate students’ research competence, especially when higher-order use was accompanied by critical thinking. R. Zhou et al. (2025) showed that ChatGPT-facilitated scaffolding improved undergraduates’ mathematical problem-solving performance and encouraged more interpretive and evaluative activity among higher-performing students. In these cases, GenAI appears to support learner agency when it functions as a resource for reflection, knowledge structuring, and strategic support rather than direct answer delivery (Zhu & Yang, 2026; R. Zhou et al., 2025).

Overall, the literature suggests that GenAI may support learner agency when it helps learners become more self-regulating, confident, evaluative, and strategically engaged. These benefits, however, are rarely automatic; they typically emerge when students remain cognitively and metacognitively involved in the task. This point becomes even clearer in the next theme, which addresses agency-eroding/offloading patterns through which GenAI may weaken agency.

### 4.3. Agency-Eroding Patterns: Cognitive Offloading, Overreliance, and Dependence

Of the 123 included studies, 108 contributed to the agency-eroding/offloading theme family. Alongside the agency-supportive patterns described above, the literature also presents a recurring and increasingly explicit account of the ways that GenAI may undermine learner agency. These agency-eroding patterns are typically discussed in terms of cognitive offloading, overreliance, dependence, cognitive outsourcing, uncritical uptake, and weakened judgement. The key concern is not that all reliance on AI is harmful but that learning may be displaced when students allow GenAI to perform the cognitive, evaluative, or authorial work that should remain part of their own learning process.

One recurring concern is that GenAI may encourage students to outsource cognitive work that would otherwise contribute to learning. Chase and Galvin (2026) described this as the risk of “outsourcing to GenAI,” warning that when AI takes over substantive thinking processes, learning validity and cognitive ownership may be compromised. Similar concerns appear in problem-solving and writing studies, where students may move from reflective use toward shortcut-oriented completion strategies. In such cases, the issue is not merely that students use assistance but that they no longer remain the primary agents of analysis, interpretation, or decision making (Chase & Galvin, 2026; Hou et al., 2025).

Several studies show that overreliance is not a uniform phenomenon but a differentiated one. Hou et al. (2025), for example, distinguished among reflective, cautious, collaborative, and thoughtless reliance behaviors. Their findings suggest that reliance becomes problematic when trust in AI is not moderated by critical thinking, and when learners shift toward thoughtless use. Zheng and Wang (2026) similarly identified different patterns of cognitive dissonance in GenAI-supported EFL learning, including efficiency–capacity dissonance, instrumental–traditional dissonance, and trust–reliance dissonance. These categories show that learners often experience tension between productivity gains and fears of losing competence, authenticity, or evaluative control. Such findings suggest that dependence is not simply a matter of how often students use GenAI but of how they resolve or fail to resolve these tensions in practice (Hou et al., 2025; Zheng & Wang, 2026).

The paradox is especially clear in studies linking GenAI use to both self-efficacy gains and dependence risks. L. Zhang and Xu (2025) found that more frequent GenAI use simultaneously enhanced students’ confidence and learning efficiency while intensifying technological dependence. Jia et al. (2025) similarly reported that GenAI dependency was associated with academic achievement through self-efficacy, but also warned that such self-efficacy could become inflated or distorted in ways resembling false competence. These studies are important because they complicate any simple positive interpretation of self-efficacy: learners may feel more capable while becoming less independently capable. From the perspective of learner agency, this means that confidence alone cannot be taken as evidence of empowerment (L. Zhang & Xu, 2025; Jia et al., 2025).

Agency-eroding/offloading patterns are also visible in studies of writing and authorship. Tekir (2026) found that frequent reliance on GenAI in EFL writing was associated with reduced originality, weaker critical reasoning, and lower authorial control, while more selective use preserved stronger personal voice and metacognitive engagement. Likewise, mixed-method studies of learning agency report that although students may experience improved self-regulated learning propensities, they may also reproduce AI-generated content uncritically or rely on AI-generated responses without sufficient evaluation. These findings indicate that agency erosion often occurs not through overt misuse alone but through subtle shifts in who is generating ideas, making judgements, and owning the resulting work (Tekir, 2026; L. Xia et al., 2026).

In sum, the agency-eroding/offloading pattern in the literature can be understood as a movement from supported learning toward displaced learning. GenAI may become problematic when it reduces the learner’s need to plan, monitor, verify, and decide. This risk does not cancel out the technology’s educational potential, but it does show that cognitive offloading, overreliance, and dependence are important for understanding how learner agency may be weakened in AI-supported higher education.

### 4.4. Recurring Mechanism-Related Construct Clusters Linking GenAI Use to Agency Outcomes

Of the 123 included studies, 116 contributed to the regulatory/evaluative mechanism-related construct theme family. Across the reviewed studies, GenAI is rarely presented as having direct or uniform implications for learner agency. Instead, the literature repeatedly links agency-related outcomes to interrelated psychological, behavioral, and socio-technical mechanism-related constructs. Among these, self-regulation, metacognition, critical thinking, self-efficacy, trust, feedback literacy, AI literacy, and cognitive offloading/reliance/dependence recur across the charted corpus. These constructs help organize why GenAI may serve as a scaffold for some learners and as a source of dependence for others, although the heterogeneous evidence base does not support a formal ranking of their causal importance (Xu et al., 2025; Zhu & Yang, 2026; C. Zhou & Wang, 2026).

Among these variables, self-regulation appears as a recurring overarching construct rather than a formally established single causal driver. Studies repeatedly show that when learners use GenAI in ways that involve goal setting, monitoring, evaluation, and strategy revision, they are more likely to benefit from AI support without losing ownership of learning. Xu et al. (2025) showed that metacognitive support helped maintain and improve self-regulated learning in GenAI environments. Q. Xia (2025) further examined the dynamic relationship between self-regulated learning and deeper approaches to learning over time. These findings suggest that self-regulation is one process through which learner agency is enacted in AI-supported learning, rather than definitive evidence that it has primacy across all contexts (Xu et al., 2025; Q. Xia, 2025).

Metacognition and metacognitive awareness form a closely related construct cluster. Teng (2025) found that learners’ metacognitive awareness strongly shaped whether they copied from ChatGPT or used it as a resource for reflection and improvement. Urban et al. (2025) showed that metacognitive accuracy and epistemic beliefs influenced whether students correctly integrated or incorrectly adopted ChatGPT-generated information in academic writing. Yao et al. (2025) similarly identified planning, monitoring, evaluating, information management, and debugging as central metacognitive strategies used by postgraduate students when employing ChatGPT for academic writing. This interpretation is also consistent with recent work warning that GenAI-supported learning can foster metacognitive laziness when learners accept fluent output without sustained monitoring or evaluation. Together, these studies indicate that metacognition shapes whether AI outputs are treated as raw material for thinking or as authoritative content to be adopted with minimal scrutiny (Teng, 2025; Urban et al., 2025; Yao et al., 2025; Fan et al., 2025).

Another recurring mechanism-related construct is critical thinking. In several studies, critical thinking functions as either a mediator, a moderator, or a protective capacity against uncritical AI uptake. Hou et al. (2025) showed that critical thinking promoted reflective and cautious reliance while buffering the effect of trust on thoughtless use. Zhu and Yang (2026) found that critical thinking mediated the relationship between GenAI use and graduate students’ research competence. Tian and Xiang (2026) further reported that critical thinking, together with self-regulation, was an important predictor in their model of effective GenAI use. These findings collectively suggest that critical thinking is not simply an educational outcome threatened by AI but a recurring construct that shapes how AI is used in the first place (Hou et al., 2025; Tian & Xiang, 2026; Zhu & Yang, 2026).

Self-efficacy also emerges as a recurring bridge construct, although the literature suggests that its role is ambivalent. On the positive side, self-efficacy often enables stronger engagement, more active use of feedback, and greater persistence in AI-supported tasks. On the negative side, self-efficacy may coexist with technological dependence or become inflated when learners mistake AI-assisted success for independent competence. This dual role is clear across studies of engagement, research competence, and dependence, suggesting that self-efficacy should be interpreted cautiously in GenAI environments: it can support agentic learning, but only when accompanied by reflection and evaluative judgement (Q. Zhang et al., 2025; L. Zhang & Xu, 2025; Jia et al., 2025).

Finally, trust and feedback literacy function as important connecting constructs between technological affordances and learner response. Q. Zhang et al. (2025) showed that responsiveness and personalization did not influence engagement directly in the same way; instead, trust and AI learning self-efficacy played mediating roles. C. Zhou and Wang (2026) showed that generative AI acceptance improved writing feedback literacy by increasing self-efficacy and reducing anxiety. Zhan and Yan (2025) also found that students’ engagement with ChatGPT feedback was often limited by shallow behavioral participation, which points to the need for stronger prompt-based, evaluative, and metacognitive feedback literacy. Taken together, these studies show that learners’ agency in AI-supported contexts depends not only on access to AI support but also on whether they trust it appropriately, can use it competently, and can evaluate and apply its feedback selectively (Q. Zhang et al., 2025; C. Zhou & Wang, 2026; Zhan & Yan, 2025).

### 4.5. Boundary Conditions and Pedagogical Responses

A final major theme in the literature concerns the conditions under which GenAI is more likely to enhance rather than erode learner agency. The reviewed studies suggest that GenAI does not operate in isolation; its effects depend on how it is pedagogically embedded, how learners are guided to use it, and whether the learning design preserves reflection, evaluation, and responsibility. In other words, the field increasingly treats GenAI not as a self-sufficient instructional solution but as a technology whose educational value depends on surrounding supports and constraints.

One recurring finding is that scaffolded use is more beneficial than direct-answer use in the studies that directly compared or discussed these designs. Studies of guidance-based, Socratic, and scaffolded GenAI designs indicate that learners are more likely to remain active interpreters when AI support provides hints, prompts, feedback, or intermediate guidance rather than final answers. In these designs, GenAI is portrayed as more educationally productive when embedded in structures that require students to inspect, revise, and justify their reasoning rather than passively accept system output.

A second boundary condition concerns metacognitive prompting and reflective support. Joo et al. (2026) found that metacognitive prompts in GenAI-supported online self-regulated learning reduced cognitive load and improved performance and self-efficacy. Reflection journals have also been shown to function as useful scaffolds in GenAI-assisted writing. D. Zhang et al. (2025) reported that structured and semi-structured reflection journals improved postgraduate students’ GenAI literacy, especially in relation to critical evaluation, ethics, and autonomy. These findings suggest that reflection should not be treated as an optional add-on to AI use; rather, it is one of the pedagogical mechanisms through which learner agency is preserved (Joo et al., 2026; D. Zhang et al., 2025).

A third boundary condition is AI literacy and feedback literacy development. Several studies show that learners require more than technical familiarity to use GenAI well. They must also develop the capacity to judge output quality, recognize risk, formulate productive prompts, and use feedback selectively. Studies of writing feedback literacy, engagement with ChatGPT feedback, CEFR-based mediation, learner autonomy, and GenAI literacy all point to the same conclusion: students are more likely to use GenAI agentically when they understand not only how to operate the tool but how to question it, verify it, and position themselves in relation to it. In this sense, AI literacy is less about technological comfort than about preserving human judgement within AI-supported learning (Zhan & Yan, 2025; C. Zhou & Wang, 2026; Lukešová & Jennings, 2025; D. Zhang et al., 2025).

Finally, several studies suggest that teacher guidance and human mediation remain important, even in highly AI-supported environments. Research on academic help-seeking, feedback use, and collaborative tasks suggests that students may be drawn to GenAI because of fluency, immediacy, and convenience, but these same qualities may increase the risk of shallow trust or premature closure unless pedagogical structures require verification and reflection. Consequently, a recurring practical implication across the literature is not that educators should reject GenAI but that they should design for augmentation rather than replacement. This means keeping learners in roles that require interpretation, evaluation, decision making, and accountability, even when AI support is available (Wang & Zhang, 2026; Zhu & Yang, 2026).

Taken together, these studies show that the difference between agency-enhancing GenAI use and agency-eroding GenAI use often lies in the surrounding design. Guidance-based interaction, reflective prompts, scaffolded feedback, AI literacy development, and continued human mediation all function as boundary conditions that shape whether GenAI becomes a cognitive scaffold or a cognitive substitute. This conclusion sets the stage for the Section 5, which integrates these themes into an interpretive dual-pattern account of GenAI, cognitive offloading, and learner agency in higher education.

The framework illustrates the authors’ configurative interpretation of the charted literature. It shows how GenAI use in higher education is associated with recurring psychological, behavioral, and socio-technical mechanism-related constructs, including self-regulation, metacognition, critical thinking, self-efficacy, trust, feedback literacy, AI literacy, and cognitive offloading/reliance/dependence. These mechanism-related constructs are interpreted as contributing to either agency-supportive or agency-eroding patterns of GenAI use. Pedagogical conditions such as scaffolding, metacognitive prompting, AI literacy, feedback literacy, and teacher guidance shape whether GenAI functions as augmentation rather than replacement. The framework is not intended as a statistically tested causal model.

**Figure 2:**
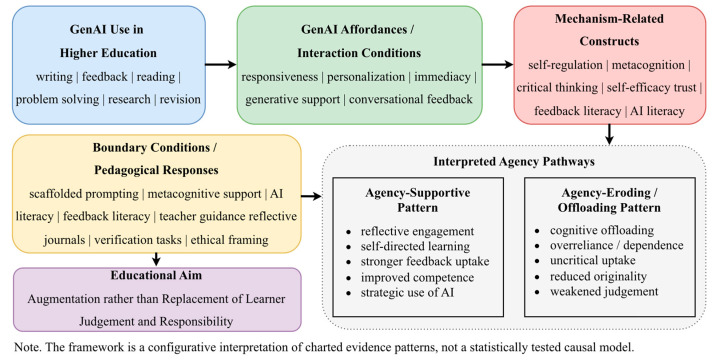
Interpretive framework linking GenAI affordances, mechanism-related constructs, agency-related patterns, and pedagogical boundary conditions.

Table 2 summarizes the five major themes identified in the review, the number of charted studies contributing to each theme family, the evidence base, the main constructs, and the interpretation. Note. Theme-family counts are not mutually exclusive because a single included study could contribute to more than one theme family. Figure 2 adds distinct value by showing how those themes relate to one another as an interpretive framework: GenAI affordances are filtered through mechanism-related constructs and pedagogical boundary conditions, producing agency-supportive or agency-eroding patterns.

## 5. Discussion

The findings of this review suggest that the educational significance of GenAI in higher education is usefully interpreted not through a simple benefits-versus-risks framing but through an interpretive dual-pattern account. Across the reviewed literature, GenAI is often portrayed as a technology that can both support and weaken learner agency. Agency-supportive patterns involve self-regulation, confidence, feedback engagement, and task performance. Agency-eroding patterns involve cognitive offloading, technological dependence, uncritical reproduction, and the gradual displacement of learners’ own judgement. Importantly, these patterns are not mutually exclusive. Several studies indicate that they may coexist within the same learning process, producing gains in perceived capability at the same time as losses in independent cognitive ownership.

One contribution of the present review is therefore to clarify why the literature often appears contradictory. Much of the inconsistency across studies does not arise because some researchers are “right” and others are “wrong” but because they examine different layers of the same phenomenon. Studies focusing on engagement, confidence, or performance often capture the immediate functional affordances of GenAI, such as responsiveness, personalization, and real-time support. By contrast, studies emphasizing dependence, overreliance, or erosion of originality often capture the longer-term redistribution of cognitive responsibility between learner and system. When these layers are considered together, a more coherent picture emerges: GenAI may improve the efficiency and subjective ease of learning while simultaneously altering the conditions under which learners develop autonomy, evaluative judgement, and sustained independent capability.

From this perspective, a major theoretical implication of the review is that learner agency can serve as a useful organizing construct for future GenAI research in higher education. Existing work on learning agency provides a basis for this move. The literature reviewed here shows that agency can be understood through key abilities, active actions, and essential mental characteristics, and also through concrete forms of engagement such as receptive, resistive, resourceful, and reflective activity. Taken together, these perspectives suggest that agency is not simply a downstream outcome of AI use. Rather, it is a meaningful way to judge whether AI-supported learning remains genuinely educational. If learners still set goals, monitor progress, evaluate outputs, revise strategically, and act responsibly, GenAI may be said to support agency. If these functions are increasingly ceded to the system, then even apparently successful AI use may mask the weakening of agency.

A second major implication concerns the role of self-regulation and metacognition. Across the reviewed studies, self-regulation appears as a recurring mechanism-related construct through which GenAI becomes either a scaffold or a substitute. When students engage in planning, monitoring, self-evaluation, verification, and strategic revision, GenAI is more likely to support deeper participation and reflective learning. When these regulatory capacities are weak, the technology is more likely to become a shortcut that narrows rather than expands learners’ roles in the learning process. This pattern suggests that the key issue is not AI access itself but whether learners possess and enact the metacognitive resources needed to govern that access. Put differently, the central question is not “Do students use GenAI?” but “Can they still regulate what that use means for their learning?”

The review also suggests that self-efficacy is a more ambivalent construct in AI-supported learning than has often been assumed. In traditional educational psychology, higher self-efficacy is usually interpreted as an unambiguously positive indicator of learner capacity. However, the GenAI literature complicates this assumption. Several studies indicate that AI-supported success can enhance students’ confidence and efficiency while simultaneously strengthening dependence on AI systems. In such cases, self-efficacy may reflect not only the learner’s own strengthened competence but also the ease of completing tasks with external cognitive support. This distinction is crucial. If self-efficacy grows alongside reduced independent problem-solving capability, then it may represent a form of supported confidence rather than autonomous competence. The literature on dependence and false self-efficacy therefore suggests that future research should be more cautious in interpreting positive self-efficacy effects in AI-supported contexts.

Another important conclusion is that cognitive offloading should not be conceptualized as uniformly harmful. One theoretically important development in the recent literature is the argument that strategic offloading may, under certain conditions, enhance rather than undermine deep learning. In particular, work on pedagogical partnerships with GenAI suggests that offloading routine or lower-order operations can free cognitive resources for higher-order reflection, provided that learners remain vigilant, evaluative, and responsible for the broader task. This is an important refinement of earlier offloading theory. Rather than treating all delegation as evidence of passivity, the emerging literature suggests that the educational value of offloading depends on what is delegated, why it is delegated, and whether the learner remains the final arbiter of meaning, quality, and direction. Strategic delegation may support transformation; uncritical delegation may erode agency.

This distinction can be strengthened by connecting cognitive offloading to the broader literature on technological cognition. Federico et al. (2026) conceptualize technological cognition as an integrated system through which humans create, understand, and use technologies by coordinating causal or technical reasoning, semantic cognition, visuospatial processing, motor control, and social learning. Although their account is not specific to GenAI in higher education, it provides a useful architecture-level explanation for why technology use can either extend cognition or displace important cognitive operations. In GenAI-supported learning, strategic offloading may be agency-preserving when learners continue to perform the core operations of goal setting, causal or epistemic reasoning, verification, and evaluative judgement, while using AI as an external support. Maladaptive offloading becomes more likely when these operations are transferred to an opaque system and the learner mainly accepts fluent output without reconstructing, testing, or integrating the reasoning behind it (Federico et al., 2026).

Bluet et al. (2025) provide complementary neuroimaging evidence that observing tool-making and teaching recruits a technical-reasoning network, while teaching also engages social-cognitive processes that guide attention to relevant technical steps. This evidence is not directly about digital GenAI tools, but it helps clarify the educational issue at stake: genuine learning with technology requires learners to remain engaged in the reasoning processes that make tool use intelligible. The agency-eroding/offloading pattern identified in this review can therefore be reframed as offloading-as-displacement: GenAI becomes agency-eroding when it replaces the learner’s technical, epistemic, or evaluative reasoning rather than supporting those processes. Conversely, augmentation-oriented pedagogy can be understood as a way of keeping technological cognition active by requiring students to inspect prompts, compare outputs, justify revisions, and explain why AI suggestions are accepted or rejected (Bluet et al., 2025; Federico et al., 2026).

This distinction between strategic and maladaptive offloading helps explain why some studies report positive outcomes from AI support while others warn of dependence and reduced originality. Strategic offloading seems most likely when AI is embedded within pedagogical structures that require students to question, verify, revise, and make explicit decisions. Maladaptive offloading, by contrast, becomes more likely when AI is used primarily for speed, answer generation, or frictionless completion. This suggests that learner agency is shaped not only by the availability of AI but by the task ecology surrounding its use. When GenAI is aligned with reflective demands, it may augment agency; when aligned primarily with efficiency demands, it may encourage displacement of agency.

A further implication of the review is that trust should be understood as a calibrated rather than uniformly desirable state. The literature often shows that trust can facilitate engagement, acceptance, and willingness to use AI-supported learning tools. Yet high trust without critical oversight can also contribute to overreliance and shallow uptake. This tension is especially visible in studies where trust and self-efficacy mediate the effects of technological features on engagement, while risk perception weakens the trust-acceptance link. The broader lesson is that educationally productive trust is not blind trust but trust that remains coupled with verification, skepticism, and epistemic accountability. In other words, what students need is not simply more trust in GenAI but more appropriately calibrated trust.

These findings carry direct pedagogical implications. A recurring implication across the charted literature is that GenAI should be designed and used as augmentation rather than replacement. This principle appears across studies of scaffolding, dialogic feedback, metacognitive prompts, reflection journals, and guidance-based learning. Reported learning benefits tend to occur when GenAI is used to provide hints, alternative perspectives, feedback for revision, or support for evaluative reflection, rather than to bypass student thinking altogether. In practice, this means that higher education instructors should avoid designing tasks in which AI can simply produce final answers with minimal human judgement. Instead, tasks should require students to justify prompt choices, compare AI outputs with other sources, reflect on why they accepted or rejected suggestions, and revise on the basis of explicit reasoning. When AI use is embedded within these reflective loops, the literature suggests that learner agency is more likely to be preserved.

The review also highlights the importance of AI literacy and feedback literacy as pedagogical priorities. Students need more than operational familiarity with GenAI tools. They need to develop evaluative judgement, risk awareness, metacognitive awareness, and the ability to interpret AI-generated feedback selectively. This is especially important in writing and research contexts, where the boundary between assistance and substitution can become blurred. Educational responses should therefore focus not only on whether students are “allowed” to use AI but on whether they are being taught to use it in ways that preserve authorship, critical reasoning, and responsibility.

At the level of future research, several directions follow from this review. First, the field would benefit from more studies that directly measure learner agency rather than relying only on adjacent variables such as acceptance, intention, or satisfaction. Second, more longitudinal and mixed-methods work is needed to understand how agency-enhancing and agency-eroding processes unfold over time. Third, future studies should more clearly distinguish strategic offloading from overreliance, and should not assume that all AI delegation has the same educational meaning. Finally, more research is needed across disciplinary and cultural contexts, since recent evidence suggests that the same dual-pathway structure may be broadly robust while still being shaped by local educational traditions and task demands.

Overall, the discussion of this review points to a cautious conclusion: GenAI does not simply support or undermine higher education learning. Rather, it redistributes the conditions under which learner agency is enacted. Whether that redistribution becomes empowering or corrosive depends on the learner’s regulatory capacities, the calibration of trust and confidence, and the pedagogical structures surrounding AI use. A productive future for GenAI in higher education will therefore not come from maximizing automation but from designing environments in which learners continue to think, judge, revise, and take responsibility while working with AI.

## 6. Implications

### 6.1. Pedagogical Implications

The findings of this review suggest that the central pedagogical challenge is no longer whether GenAI should be present in higher education but how it should be pedagogically positioned. The literature often indicates that GenAI is more beneficial when it is used as a form of augmentation rather than replacement. In practical terms, this means that GenAI should not simply be treated as an answer-generating tool that removes difficulty from learning tasks. Instead, it should be integrated in ways that preserve learners’ responsibility for interpretation, evaluation, revision, and decision making. Studies on pedagogical partnerships, research competence, and writing support indicate that the educational value of GenAI depends on whether students remain the active agents of meaning making rather than outsourcing that role to the system (Wang & Zhang, 2026; Zhu & Yang, 2026).

A first implication, therefore, is that instructors should prioritize scaffolded AI use over direct-answer use. Positive outcomes in the reviewed literature were commonly associated with learning designs in which GenAI provided hints, prompts, feedback, or intermediate support rather than fully formed solutions. Guidance-based designs, Socratic questioning, metacognitive prompting, CEFR-mediated language tasks, and AI-facilitated scaffolding appear more conducive to agency-preserving learning than unrestricted answer delivery. These approaches keep students cognitively engaged and make it more difficult for them to drift into passive acceptance or superficial completion (Lee et al., 2024; Joo et al., 2026; Lukešová & Jennings, 2025; Sun et al., 2026; R. Zhou et al., 2025).

A second implication concerns the explicit teaching of self-regulation, metacognition, and critical evaluation. The reviewed studies repeatedly show that these are not secondary skills in AI-supported learning; they are the conditions under which GenAI becomes educationally productive. Students need structured opportunities to set goals, monitor their own use of AI, compare alternative outputs, verify accuracy, justify acceptance or rejection of AI suggestions, and reflect on how AI support affects their own thinking. Reflection journals, dialogic feedback routines, and tasks that require explanation of revision choices are especially promising because they turn AI use into an object of reflection rather than a hidden background process (Zhan & Yan, 2025; D. Zhang et al., 2025; C. Zhou & Wang, 2026).

A third pedagogical implication is the need to cultivate AI literacy as evaluative literacy, not merely technical familiarity. The literature suggests that students appear more likely to benefit from GenAI when they understand not only how to use the tool but also how to question it. This includes recognizing limitations, detecting risk, calibrating trust, and distinguishing support from substitution. Instructors should therefore design learning activities that require students to evaluate AI-generated content against disciplinary standards, prior knowledge, human feedback, and alternative sources. In writing and research contexts in particular, AI literacy should be closely linked to authorship, evidence use, and responsible academic practice (Qi et al., 2025; Zhu & Yang, 2026).

A fourth implication is that feedback literacy deserves particular attention in the AI era. Several studies show that students may interact with AI-generated feedback at a shallow level unless they are taught how to request, interpret, evaluate, and act on that feedback. This means that higher education institutions should not assume that AI feedback automatically leads to revision or learning improvement. Rather, students need explicit support in developing evaluative judgement, emotional reflexivity, and selective uptake. Feedback literacy should therefore be treated as a core educational outcome in AI-supported learning, especially in writing-intensive and research-intensive programs (Zhan & Yan, 2025; C. Zhou & Wang, 2026).

Finally, the review indicates that human mediation remains essential. Even when GenAI provides highly responsive and personalized support, teachers still play a crucial role in shaping the conditions under which students use AI reflectively and responsibly. More pedagogically productive environments are not those in which teachers withdraw in favor of automation but those in which instructors guide students in using GenAI as a reflective partner, a source of challenge, and a scaffold for deeper learning. In this sense, the goal is not to minimize the teacher’s role but to reconfigure it toward designing tasks, prompting reflection, and preserving learner agency in AI-mediated education (Smirnova, 2025; Wang & Zhang, 2026).

### 6.2. Implications for Future Research

The review also has several implications for future research. First, the field would benefit from more studies that directly conceptualize and measure learner agency rather than relying only on adjacent constructs such as intention to use, acceptance, satisfaction, or performance. Recent work has begun to offer scale-based and framework-based approaches to learner agency, but the concept is still unevenly operationalized across studies. More explicit use of agency as a central construct would allow stronger comparison across contexts and improve the theoretical coherence of the field (L. Xia et al., 2025; Yang et al., 2024).

Second, future studies should more clearly distinguish between strategic cognitive offloading and maladaptive overreliance. A major contribution of the recent literature is the suggestion that not all delegation to AI is harmful. However, many studies still treat dependence, offloading, and AI assistance as if they were conceptually interchangeable. More fine-grained work is needed to identify what kinds of delegation are compatible with learner agency and what kinds undermine it. This distinction is essential if the field is to move beyond generalized anxiety about AI and toward more precise explanations of learning processes (Wang & Zhang, 2026; Hou et al., 2025).

Third, the field would benefit from more longitudinal, mixed-methods, and behavior-trace research. Much of the existing literature remains cross-sectional and self-report-based. While such studies have established important patterns, they provide limited insight into how agency-enhancing and agency-eroding effects evolve over time. Longitudinal and mixed-methods studies would make it possible to examine how students’ trust, self-regulation, feedback use, and dependence develop across sustained interaction with GenAI, and whether short-term gains in confidence translate into long-term gains in autonomous competence (Q. Xia, 2025; L. Xia et al., 2026).

Fourth, more comparative research is needed across disciplines, learner populations, and cultural contexts. The reviewed literature spans writing, language learning, research training, problem solving, and other domains, but some areas remain much more heavily represented than others. There is also a strong concentration in Chinese and EFL-related higher education settings. Although this concentration has generated valuable insights, broader disciplinary and contextual diversity would improve the generalizability of future findings and reveal whether, and how, the same dual-pattern interpretation holds across different educational traditions, digital-divide conditions, and epistemic practices (C. Zhang et al., 2026; Zheng & Wang, 2026).

## 7. Limitations

This review should be interpreted in light of several limitations. First, the review focused on English-language peer-reviewed or scholarly journal literature published from 2022 to 24 June 2026. Relevant work published in other languages, books, theses, conference proceedings, institutional reports, or other grey literature may not have been captured. As a result, the present synthesis should be understood as a structured mapping of a substantial and reviewable segment of the literature rather than an exhaustive representation of all available knowledge on the topic.

Second, the review concentrated specifically on higher education contexts and excluded studies focused primarily on teachers, faculty adoption, institutional implementation, technical system development, clinical populations, and K-12 education unless they offered direct relevance to student learning processes. This decision strengthened conceptual focus, but it also means that broader ecosystem-level factors shaping GenAI use in universities may be underrepresented in the synthesis.

Third, the literature included in the review is methodologically heterogeneous, covering surveys, experiments, qualitative studies, mixed-methods designs, behavioral analyses, scale-development studies, reviews/evidence syntheses, and conceptual/framework papers. This diversity is one reason a scoping review was appropriate, but it also means that the conclusions are conceptual and interpretive rather than causal in a narrow statistical sense. No formal critical appraisal or risk-of-bias assessment was conducted; therefore, the review avoids treating all evidence types as equivalent and does not claim that recurring mechanisms have been established with equal empirical strength across all contexts.

Fourth, a large proportion of the studies reviewed were concentrated in language-learning, writing, and Chinese/EFL higher education settings. This concentration reflects the current state of the field and provides valuable insight into writing- and feedback-intensive forms of GenAI use, but it limits the generalizability of the dual-pattern framework. The relative weight of agency support, agency erosion, offloading, dependence, and self-regulatory constructs may differ in underrepresented areas such as laboratory-based STEM education, design education, professional education, and non-EFL disciplinary contexts.

Fifth, although the revised search strategy used six database sources and searched GenAI terms in title, abstract, keyword, topic, subject, NOFT, or database-equivalent fields, it still used database filters for English-language peer-reviewed or scholarly journal literature and a 2022-onward publication window. These limits were applied for conceptual and practical reasons, but they may have excluded relevant early conceptual work on AI, automation, or cognitive offloading that did not use current GenAI terminology. In addition, the GenAI block emphasized ChatGPT, generative AI, GenAI, large language model, LLM, GPT, GPT-3, and GPT-4 terms rather than every product name or broader conversational-agent label. Studies indexed only under terms such as Gemini, Bard, Claude, Copilot, AI chatbot, conversational agent, AI assistant, or foundation model may therefore have been missed if they did not also include the searched GenAI/LLM/ChatGPT/GPT terminology.

Sixth, the full-text stage depended on reports that could be retrieved and converted for screening. Of the 717 reports sought, 26 were not retrieved despite reasonable access attempts through institutional database links, publisher pages, DOI landing pages, interlibrary or library-accessible options, and other accessible full-text routes. These unretrieved reports included records for which full text was not accessible through the authors’ institutional subscriptions at the time of screening. The retrieval decisions were documented in the screening records, and bibliographic metadata for the 123 included studies were subsequently verified against the original database exports. The supplementary charting matrix is therefore intended to support transparency and auditability while recognizing that the review remains limited by full-text availability, database indexing coverage, institutional subscription coverage, and copyright restrictions on redistribution of source articles.

Finally, many of the included studies were based on cross-sectional self-report data, which limits the ability of the underlying literature to capture how learner agency, dependence, and offloading develop over time. Accordingly, although this review proposes a dual-pattern interpretation, the temporal dynamics of that interpretation remain to be tested more rigorously through longitudinal and multimodal research.

## 8. Conclusions

This scoping review examined how the literature has conceptualized the relationships among generative AI, cognitive offloading, and learner agency in higher education. Based on 123 included studies retained from a six-database search, the review shows that GenAI cannot be adequately understood through a simple positive-or-negative lens. Instead, the literature points to an interpretive dual-pattern structure. One pattern suggests that GenAI may support learner agency by supporting self-regulation, confidence, feedback engagement, and strategic participation in learning. The other suggests that GenAI may weaken learner agency by encouraging cognitive offloading, overreliance, technological dependence, and uncritical uptake of AI-generated output.

A key conclusion of the review is that learner agency offers a useful organizing framework for interpreting these tensions. The central question is not whether students use GenAI but whether they retain the capacity to direct, monitor, evaluate, and take responsibility for that use. In this sense, learner agency serves as both an analytic construct and a normative educational criterion: it helps explain variation in outcomes and it identifies what should be preserved in AI-mediated learning.

The review also indicates that self-regulation and metacognition are recurring mechanism-related constructs through which GenAI becomes either a scaffold or a substitute. When learners continue to set goals, verify outputs, reflect on feedback, and exercise evaluative judgement, GenAI is more likely to function as augmentation. When these processes weaken, the same technology may begin to displace rather than support thinking. Trust, self-efficacy, anxiety, feedback literacy, AI literacy, and the broader architecture of technological cognition further shape this process by influencing how students interpret and act on AI-supported learning opportunities.

Overall, the findings suggest that the future educational value of GenAI in higher education will depend less on how capable the technology becomes and more on how effectively learning environments preserve human judgement, reflective participation, and responsibility. A productive path forward is therefore not to maximize automation for its own sake but to design GenAI-supported education in ways that keep learners actively involved in thinking, evaluating, revising, and deciding. In that sense, the fundamental challenge of the AI era is not simply teaching students to use GenAI but ensuring that they remain agentic learners while doing so.

## Figures and Tables

**Table 1:** Characteristics of the included studies (n = 123).

Characteristic	Count/Description
Included studies	n = 123 included studies; complete study-level metadata, coding fields, and citations are provided in Supplementary Table S1.
Publication years	2024 (n = 5), 2025 (n = 34), and 2026 (n = 84).
Evidence categories	Empirical studies (n = 115), review/evidence-synthesis articles (n = 7), and conceptual/framework source (n = 1). Main method labels: experimental/intervention/quasi-experimental (n = 33), mixed-methods/Q-methodology (n = 25), quantitative survey/correlational (n = 24), qualitative (n = 20), scale development/validation (n = 6), review/evidence synthesis (n = 6), scoping review (n = 1), conceptual/framework (n = 1), and empirical study not further classifiable from available fields (n = 7).
Retained source after deduplication	Scopus (n = 67), EBSCOhost Education Source (n = 25), APA PsycInfo via ProQuest (n = 15), EBSCOhost ERIC (n = 8), ProQuest Education Collection via ProQuest Social Science Premium Collection (n = 5), EBSCOhost combined/exported source (n = 2), and Web of Science Core Collection (n = 1). Duplicate studies were assigned to the source retained after deduplication.
Country/region signals	Non-mutually exclusive signals most often identified China (n = 58) and the United States (n = 46), followed by Malaysia (n = 8), Indonesia (n = 7), Türkiye/Turkey (n = 6), and Korea, Australia, and the United Kingdom (n = 5 each). Country/region was not clearly identifiable for 15 studies.
Educational-context signals	Research/postgraduate learning (n = 100), discipline-specific higher education (n = 77), language/EFL/ESL (n = 71), writing/feedback (n = 70), STEM/problem solving (n = 70), and health/medical education (n = 10).
Participant/task signals	Participant signals: undergraduate/university students (n = 91), EFL/ESL learners (n = 61), postgraduate/graduate students (n = 40), health/medical students (n = 8), and general higher education learners/students (n = 6). Task/activity signals: problem solving/programming/STEM (n = 48), research/thesis/postgraduate tasks (n = 45), writing/revision/feedback (n = 40), language learning/communication (n = 32), assessment/reflection/metacognitive tasks (n = 30), creative/design tasks (n = 25), and reading/summarizing/information evaluation (n = 25).
Construct/theme signals	Signals were counted only when a construct was substantively discussed, measured, operationalized, or used to interpret findings. Agency/autonomy signals appeared in 121 studies, offloading/reliance/dependence signals in 122 studies, and mechanism-related signals in 123 studies. Theme-family counts were: agency/autonomy conceptualization (n = 86), agency-supportive patterns (n = 118), agency-eroding/offloading patterns (n = 108), regulatory/evaluative mechanism-related constructs (n = 116), and boundary conditions/pedagogical responses (n = 120).
Full-text exclusions	Supplementary Table S2 reports all 568 full-text exclusions with final reasons after independent full-text rescreening and fourth-author adjudication.

Note: These signal counts partly reflect the review’s eligibility criteria, which required substantive relevance to learner agency, offloading/reliance/dependence, or closely related mechanism-oriented learning constructs.

**Table 2:** Theme-evidence matrix for the included studies.

Theme Family	n and Evidence Base	Main Constructs	Interpretation
Learner agency/autonomy conceptualization	n = 86; agency/autonomy signals in 121/123. Evidence mainly empirical (n = 81), plus review/evidence syntheses (n = 5).	Learner agency; autonomy; self-directed learning; authorship; voice; intentionality; responsibility.	Agency is mapped as multidimensional and context-sensitive, not inferred from tool use alone.
Agency-supportive patterns	n = 118; coded through self-regulation, self-efficacy, feedback literacy, engagement, and scaffolding. Evidence: empirical (n = 111), reviews/syntheses (n = 6), conceptual/framework (n = 1).	Self-regulation; self-efficacy; feedback literacy; reflective engagement; scaffolded learning.	GenAI may support agency when learners remain involved in planning, monitoring, evaluating, and revising.
Agency-eroding/offloading patterns	n = 108; offloading/reliance/dependence signals in 122/123. Evidence: empirical (n = 100), reviews/syntheses (n = 7), conceptual/framework (n = 1).	Cognitive offloading; overreliance; dependence; cognitive outsourcing; uncritical uptake; false self-efficacy.	Agency may be weakened when learners transfer judgement, idea generation, verification, or authorship to GenAI without reflective oversight.
Regulatory/evaluative mechanism-related constructs	n = 116; mechanism-related signals in all 123 studies and consolidated during configurative synthesis. Evidence: empirical (n = 108), reviews/syntheses (n = 7), conceptual/framework (n = 1).	Self-regulation; metacognition; critical thinking; trust; self-efficacy; AI literacy; feedback literacy; evaluative judgement.	Constructs are treated as recurring clusters in a heterogeneous literature, not as a formally ranked causal hierarchy.
Boundary conditions and pedagogical responses	n = 120; coded through scaffolding, guidance, prompting, reflection, AI literacy, feedback literacy, and teacher mediation. Evidence: empirical (n = 112), reviews/syntheses (n = 7), conceptual/framework (n = 1).	Scaffolding; metacognitive prompts; dialogic feedback; reflection journals; calibrated trust; teacher guidance.	Pedagogical embedding shapes whether GenAI functions as augmentation or replacement.

## Data Availability

No new primary empirical data were collected for this study. The review generated and analyzed secondary review data, including search records, deduplication decisions, title/abstract screening decisions, full-text eligibility decisions, exclusion reasons, charting fields, and thematic codes. The complete charting matrix of the 123 included studies is provided as Supplementary Table S1. Full-text exclusion reasons and screening reliability information are provided as Supplementary Table S2. The file manifest linking retained full texts to included study records can be made available by the corresponding authors upon reasonable request, subject to copyright restrictions on full-text articles. Complete citations for all 123 included studies are provided in Supplementary Table S1; the manuscript reference list cites the included studies and external theoretical/methodological sources that are discussed directly in the main text.

## Supplementary Materials

The following supporting information can be downloaded at: https://www.mdpi.com/article/10.3390/bs16071150/s1: Table S1: Complete charting matrix of the included studies (n = 123); Table S2: Full-text exclusion reasons and screening reliability information.

## Appendix A. PRISMA-ScR Checklist

This review was reported with reference to the PRISMA extension for Scoping Reviews (PRISMA-ScR). Table A1 indicates where each checklist item is addressed in the manuscript.

**Table A1:** PRISMA-ScR checklist and location of reported items in the manuscript.

Section	Item No.	PRISMA-ScR Item	Reported in Manuscript
**Title**	1	Identify the report as a scoping review.	Title
**Abstract**	2	Provide a structured summary that includes background, objectives, eligibility criteria, sources of evidence, charting methods, results, and conclusions.	Abstract
**Introduction**	3	Describe the rationale for the review in the context of what is already known and explain why the review questions are suitable for a scoping review approach.	Section 1. Introduction
**Introduction**	4	State the objectives or questions of the review with reference to key elements such as population, concepts, and context.	Section 1. Introduction
**Methods**	5	Indicate whether a review protocol exists and, if applicable, where it can be accessed.	Section 3.1. Review Design The review protocol was registered in INPLASY. Registration number: INPLASY202650152; DOI: 10.37766/inplasy2026.5.0152.
**Methods**	6	Specify the eligibility criteria for sources of evidence, including years considered, language, publication status, and rationale.	Section 3.3. Eligibility Criteria
**Methods**	7	Describe all information sources used in the search and the date of the most recent search.	Section 3.2. Search Strategy; Appendix B
**Methods**	8	Present the full electronic search strategy for at least one database, including any limits used.	Appendix B
**Methods**	9	Describe the process for selecting sources of evidence, including screening and eligibility assessment.	Section 3.4. Study Selection; Supplementary Table S2; screening reliability summary.
**Methods**	10	Describe the methods used to chart data from the included studies.	Section 3.5. Data Charting; Supplementary Table S1.
**Methods**	11	List and define all variables for which data were sought and any assumptions or simplifications made.	Section 3.5. Data Charting; Supplementary Table S1.
**Methods**	12	If done, describe any critical appraisal of included sources and how this information was used.	Not applicable; formal critical appraisal was not conducted
**Methods**	13	Describe the methods used to summarize and synthesize the charted data.	Section 3.6. Data Synthesis
**Results**	14	Report the number of sources screened, assessed for eligibility, and included, with reasons for exclusions at each stage, ideally using a flow diagram.	Section 3.4. Study Selection; Figure 1; Supplementary Table S2.
**Results**	15	Describe the characteristics of the included sources of evidence.	Table 1; Supplementary Table S1; Section 4.
**Results**	16	If done, present results of any critical appraisal of included sources.	Not applicable
**Results**	17	Present the relevant data that were charted from each included source of evidence.	Supplementary Table S1; Table 1 and Table 2; Section 4.
**Results**	18	Summarize and present the charting results in relation to the review questions and objectives.	Section 4.1, Section 4.2, Section 4.3, Section 4.4 and Section 4.5; Table 2; Supplementary Table S1.
**Discussion**	19	Summarize the main results, including an overview of concepts, themes, and types of evidence available, and link these to the review questions.	Section 5. Discussion
**Discussion**	20	Discuss the limitations of the scoping review process.	Section 7. Limitations
**Discussion/Conclusion**	21	Provide a general interpretation of the results with respect to the review questions and possible implications.	Section 5. Discussion; Section 6. Implications; Section 8. Conclusions
**Funding**	22	Describe sources of funding for the included sources of evidence, where available, and describe the role of funders for the scoping review.	Funding Statement

## Appendix B. Full Search Strategy

### Appendix B.1. Database and Search Scope

The literature search was conducted on 24 June 2026. To improve breadth and transparency, the updated search covered six database sources: Web of Science Core Collection, Scopus, EBSCOhost ERIC, EBSCOhost Education Source, APA PsycInfo via ProQuest, and ProQuest Education Collection via ProQuest Social Science Premium Collection. The search focused on English-language peer-reviewed or scholarly journal records published from 2022 to 24 June 2026.

The search strategy was designed to identify studies at the intersection of three conceptual domains: (1) generative AI technologies, including ChatGPT, generative AI, GenAI, large language models, LLMs, and GPT-based tools; (2) higher education contexts, including university, college, tertiary education, undergraduate, postgraduate, graduate student, university student, and college student settings; and (3) agency-, offloading-, and mechanism-related constructs, including learner agency, autonomy, self-directed learning, cognitive offloading, reliance, dependence, self-regulation, metacognition, critical thinking, self-efficacy, trust, feedback literacy, evaluative judgement/judgment, and AI literacy.

GenAI-related terms were searched in title, abstract, keyword, topic, subject, NOFT, or database-equivalent indexed fields, depending on the functionality of each database. No exclusion terms for primary education, secondary education, clinical populations, or non-student contexts were included in the database strings; these exclusions were applied during screening to reduce the risk of false negatives.

### Appendix B.2. Database Sources, Filters, and Records Retrieved

Across the six database sources, 8020 records were retrieved before deduplication: Web of Science Core Collection (n = 939), Scopus (n = 2524), EBSCOhost ERIC (n = 610), EBSCOhost Education Source (n = 1316), APA PsycInfo via ProQuest (n = 726), and ProQuest Education Collection via ProQuest Social Science Premium Collection (n = 1905). Database filters were applied to retain English-language peer-reviewed/scholarly journal records from 2022 to 24 June 2026. In Web of Science, Article, Review Article, and Early Access were selected. In Scopus, article and review-type scholarly journal records were retained. In EBSCOhost and ProQuest, peer-reviewed/scholarly journal filters and equivalent document-type restrictions were applied where available.

### Appendix B.3. Full Search Strings

Web of Science Core Collection: (TS = (ChatGPT OR “generative AI” OR “generative artificial intelligence” OR GenAI OR “large language model*” OR LLM* OR GPT OR “GPT-3” OR “GPT-4”) AND TS = (“higher education” OR universit* OR college* OR “tertiary education” OR undergraduate* OR postgraduate* OR “graduate student*” OR “university student*” OR “college student*”) AND TS = (“learner agency” OR “student agency” OR “learning agency” OR autonom* OR “self-directed learning”)) OR (TS = (ChatGPT OR “generative AI” OR “generative artificial intelligence” OR GenAI OR “large language model*” OR LLM* OR GPT OR “GPT-3” OR “GPT-4”) AND TS = (“higher education” OR universit* OR college* OR “tertiary education” OR undergraduate* OR postgraduate* OR “graduate student*” OR “university student*” OR “college student*”) AND TS = (“cognitive offload*” OR overreliance OR “over-reliance” OR reliance OR “AI reliance” OR dependenc* OR “technological dependence” OR “cognitive outsourcing” OR “automation bias”)) OR (TS = (ChatGPT OR “generative AI” OR “generative artificial intelligence” OR GenAI OR “large language model*” OR LLM* OR GPT OR “GPT-3” OR “GPT-4”) AND TS = (“higher education” OR universit* OR college* OR “tertiary education” OR undergraduate* OR postgraduate* OR “graduate student*” OR “university student*” OR “college student*”) AND TS = (“self-efficacy” OR “self efficacy” OR self-regulat* OR “self-regulated learning” OR metacognit* OR “critical thinking” OR “feedback literacy” OR trust OR “evaluative judgement” OR “evaluative judgment” OR “AI literacy”)).

Scopus: ((TITLE-ABS-KEY (ChatGPT OR “generative AI” OR “generative artificial intelligence” OR GenAI OR “large language model*” OR LLM* OR GPT OR “GPT-3” OR “GPT-4”) AND TITLE-ABS-KEY (“higher education” OR universit* OR college* OR “tertiary education” OR undergraduate* OR postgraduate* OR “graduate student*” OR “university student*” OR “college student*”) AND TITLE-ABS-KEY (“learner agency” OR “student agency” OR “learning agency” OR autonom* OR “self-directed learning”)) OR (TITLE-ABS-KEY (ChatGPT OR “generative AI” OR “generative artificial intelligence” OR GenAI OR “large language model*” OR LLM* OR GPT OR “GPT-3” OR “GPT-4”) AND TITLE-ABS-KEY (“higher education” OR universit* OR college* OR “tertiary education” OR undergraduate* OR postgraduate* OR “graduate student*” OR “university student*” OR “college student*”) AND TITLE-ABS-KEY (“cognitive offload*” OR overreliance OR “over-reliance” OR reliance OR “AI reliance” OR dependenc* OR “technological dependence” OR “cognitive outsourcing” OR “automation bias”)) OR (TITLE-ABS-KEY (ChatGPT OR “generative AI” OR “generative artificial intelligence” OR GenAI OR “large language model*” OR LLM* OR GPT OR “GPT-3” OR “GPT-4”) AND TITLE-ABS-KEY (“higher education” OR universit* OR college* OR “tertiary education” OR undergraduate* OR postgraduate* OR “graduate student*” OR “university student*” OR “college student*”) AND TITLE-ABS-KEY (“self-efficacy” OR “self efficacy” OR self-regulat* OR “self-regulated learning” OR metacognit* OR “critical thinking” OR “feedback literacy” OR trust OR “evaluative judgement” OR “evaluative judgment” OR “AI literacy”))). Filters: English; 2022–2026; Article and Review; records retrieved n = 2524.

EBSCOhost ERIC and EBSCOhost Education Source: (((TI (ChatGPT OR “generative AI” OR “generative artificial intelligence” OR GenAI OR “large language model*” OR LLM* OR GPT OR “GPT-3” OR “GPT-4”) OR AB (ChatGPT OR “generative AI” OR “generative artificial intelligence” OR GenAI OR “large language model*” OR LLM* OR GPT OR “GPT-3” OR “GPT-4”) OR SU (ChatGPT OR “generative AI” OR “generative artificial intelligence” OR GenAI OR “large language model*” OR LLM* OR GPT OR “GPT-3” OR “GPT-4”)) AND (TI (“higher education” OR universit* OR college* OR “tertiary education” OR undergraduate* OR postgraduate* OR “graduate student*” OR “university student*” OR “college student*”) OR AB (“higher education” OR universit* OR college* OR “tertiary education” OR undergraduate* OR postgraduate* OR “graduate student*” OR “university student*” OR “college student*”) OR SU (“higher education” OR universit* OR college* OR “tertiary education” OR undergraduate* OR postgraduate* OR “graduate student*” OR “university student*” OR “college student*”)) AND (TI (“learner agency” OR “student agency” OR “learning agency” OR autonom* OR “self-directed learning”) OR AB (“learner agency” OR “student agency” OR “learning agency” OR autonom* OR “self-directed learning”) OR SU (“learner agency” OR “student agency” OR “learning agency” OR autonom* OR “self-directed learning”))) OR ((TI (ChatGPT OR “generative AI” OR “generative artificial intelligence” OR GenAI OR “large language model*” OR LLM* OR GPT OR “GPT-3” OR “GPT-4”) OR AB (ChatGPT OR “generative AI” OR “generative artificial intelligence” OR GenAI OR “large language model*” OR LLM* OR GPT OR “GPT-3” OR “GPT-4”) OR SU (ChatGPT OR “generative AI” OR “generative artificial intelligence” OR GenAI OR “large language model*” OR LLM* OR GPT OR “GPT-3” OR “GPT-4”)) AND (TI (“higher education” OR universit* OR college* OR “tertiary education” OR undergraduate* OR postgraduate* OR “graduate student*” OR “university student*” OR “college student*”) OR AB (“higher education” OR universit* OR college* OR “tertiary education” OR undergraduate* OR postgraduate* OR “graduate student*” OR “university student*” OR “college student*”) OR SU (“higher education” OR universit* OR college* OR “tertiary education” OR undergraduate* OR postgraduate* OR “graduate student*” OR “university student*” OR “college student*”)) AND (TI (“cognitive offload*” OR overreliance OR “over-reliance” OR reliance OR “AI reliance” OR dependenc* OR “technological dependence” OR “cognitive outsourcing” OR “automation bias”) OR AB (“cognitive offload*” OR overreliance OR “over-reliance” OR reliance OR “AI reliance” OR dependenc* OR “technological dependence” OR “cognitive outsourcing” OR “automation bias”) OR SU (“cognitive offload*” OR overreliance OR “over-reliance” OR reliance OR “AI reliance” OR dependenc* OR “technological dependence” OR “cognitive outsourcing” OR “automation bias”))) OR ((TI (ChatGPT OR “generative AI” OR “generative artificial intelligence” OR GenAI OR “large language model*” OR LLM* OR GPT OR “GPT-3” OR “GPT-4”) OR AB (ChatGPT OR “generative AI” OR “generative artificial intelligence” OR GenAI OR “large language model*” OR LLM* OR GPT OR “GPT-3” OR “GPT-4”) OR SU (ChatGPT OR “generative AI” OR “generative artificial intelligence” OR GenAI OR “large language model*” OR LLM* OR GPT OR “GPT-3” OR “GPT-4”)) AND (TI (“higher education” OR universit* OR college* OR “tertiary education” OR undergraduate* OR postgraduate* OR “graduate student*” OR “university student*” OR “college student*”) OR AB (“higher education” OR universit* OR college* OR “tertiary education” OR undergraduate* OR postgraduate* OR “graduate student*” OR “university student*” OR “college student*”) OR SU (“higher education” OR universit* OR college* OR “tertiary education” OR undergraduate* OR postgraduate* OR “graduate student*” OR “university student*” OR “college student*”)) AND (TI (“self-efficacy” OR “self efficacy” OR self-regulat* OR “self-regulated learning” OR metacognit* OR “critical thinking” OR “feedback literacy” OR trust OR “evaluative judgement” OR “evaluative judgment” OR “AI literacy”) OR AB (“self-efficacy” OR “self efficacy” OR self-regulat* OR “self-regulated learning” OR metacognit* OR “critical thinking” OR “feedback literacy” OR trust OR “evaluative judgement” OR “evaluative judgment” OR “AI literacy”) OR SU (“self-efficacy” OR “self efficacy” OR self-regulat* OR “self-regulated learning” OR metacognit* OR “critical thinking” OR “feedback literacy” OR trust OR “evaluative judgement” OR “evaluative judgment” OR “AI literacy”)))). Filters: English; 2022–2026; peer-reviewed/scholarly journal records where available; ERIC n = 610; Education Source n = 1316.

APA PsycInfo via ProQuest and ProQuest Education Collection via ProQuest Social Science Premium Collection: ((NOFT (ChatGPT OR “generative AI” OR “generative artificial intelligence” OR GenAI OR “large language model*” OR LLM* OR GPT OR “GPT-3” OR “GPT-4”) AND NOFT (“higher education” OR universit* OR college* OR “tertiary education” OR undergraduate* OR postgraduate* OR “graduate student*” OR “university student*” OR “college student*”) AND NOFT (“learner agency” OR “student agency” OR “learning agency” OR autonom* OR “self-directed learning”)) OR (NOFT (ChatGPT OR “generative AI” OR “generative artificial intelligence” OR GenAI OR “large language model*” OR LLM* OR GPT OR “GPT-3” OR “GPT-4”) AND NOFT (“higher education” OR universit* OR college* OR “tertiary education” OR undergraduate* OR postgraduate* OR “graduate student*” OR “university student*” OR “college student*”) AND NOFT (“cognitive offload*” OR overreliance OR “over-reliance” OR reliance OR “AI reliance” OR dependenc* OR “technological dependence” OR “cognitive outsourcing” OR “automation bias”)) OR (NOFT (ChatGPT OR “generative AI” OR “generative artificial intelligence” OR GenAI OR “large language model*” OR LLM* OR GPT OR “GPT-3” OR “GPT-4”) AND NOFT (“higher education” OR universit* OR college* OR “tertiary education” OR undergraduate* OR postgraduate* OR “graduate student*” OR “university student*” OR “college student*”) AND NOFT (“self-efficacy” OR “self efficacy” OR self-regulat* OR “self-regulated learning” OR metacognit* OR “critical thinking” OR “feedback literacy” OR trust OR “evaluative judgement” OR “evaluative judgment” OR “AI literacy”))). Filters: English; 2022–2026; scholarly journals and peer-reviewed journal records where available; APA PsycInfo n = 726; ProQuest Education Collection via ProQuest Social Science Premium Collection n = 1905.

### Appendix B.4. Screening Notes

All retrieved records were exported from the six database sources and prepared for deduplication. Duplicate records across databases were removed before title and abstract screening. Title and abstract screening then retained records when they were situated in higher education, focused on GenAI or large language model-based tools, and addressed learner agency/autonomy/self-directed learning, cognitive offloading/overreliance/dependence, or closely related mechanism-oriented constructs such as self-regulation, metacognition, self-efficacy, critical thinking, feedback literacy, AI literacy, trust, or evaluative judgement.

Records were excluded during screening when they focused primarily on primary or secondary education, clinical or patient populations, non-educational contexts, teacher or institutional adoption without direct relevance to student learning processes, technical system development without learner evidence, or general technology acceptance without agency/offloading/mechanism relevance.

### Appendix B.5. Full Screening Flow

The updated search identified 8020 records. After 2693 duplicate records were removed, 5327 records were screened by title and abstract. Three authors independently screened these records, and unresolved disagreements were adjudicated by a fourth author. Title/abstract screening excluded 4610 records and retained 717 reports for retrieval. Of these, 26 reports could not be retrieved despite reasonable access attempts, including records not accessible through the authors’ institutional subscriptions or other accessible full-text routes at the time of screening. A total of 691 full-text reports were retrieved and assessed for eligibility. Full-text screening excluded 568 reports with reasons and retained 123 studies for charting and synthesis. Detailed exclusion reasons are reported in Supplementary Table S2.

### Appendix B.6. Note on Search Revision

The revised strategy differs from the earlier draft by expanding the search from one database to six database sources, moving GenAI terms beyond title-only searching, documenting exact retrieval counts by source, and reporting the deduplication, screening, retrieval, full-text eligibility, exclusion reason, and inclusion stages in a PRISMA-ScR-compatible format.
